# A survey of data element perspective: Application of artificial intelligence in health big data

**DOI:** 10.3389/fnins.2022.1031732

**Published:** 2022-10-25

**Authors:** Honglin Xiong, Hongmin Chen, Li Xu, Hong Liu, Lumin Fan, Qifeng Tang, Hsunfang Cho

**Affiliations:** ^1^Antai College of Economics and Management, Shanghai Jiao Tong University, Shanghai, China; ^2^Business School, University of Shanghai for Science and Technology, Shanghai, China; ^3^Operation Management Department, East Hospital Affiliated to Tongji University, Shanghai, China; ^4^Department of Computer Science and Engineering, East China University of Science and Technology, Shanghai, China; ^5^National Engineering Laboratory for Big Data Distribution and Exchange Technologies, Shanghai, China; ^6^Shanghai Data Exchange Corporation, Shanghai, China

**Keywords:** healthcare big data, machine learning, automatic diagnosis, healthcare informatics, data elements, artificial intelligence

## Abstract

Artificial intelligence (AI) based on the perspective of data elements is widely used in the healthcare informatics domain. Large amounts of clinical data from electronic medical records (EMRs), electronic health records (EHRs), and electroencephalography records (EEGs) have been generated and collected at an unprecedented speed and scale. For instance, the new generation of wearable technologies enables easy-collecting peoples’ daily health data such as blood pressure, blood glucose, and physiological data, as well as the application of EHRs documenting large amounts of patient data. The cost of acquiring and processing health big data is expected to reduce dramatically with the help of AI technologies and open-source big data platforms such as Hadoop and Spark. The application of AI technologies in health big data presents new opportunities to discover the relationship among living habits, sports, inheritances, diseases, symptoms, and drugs. Meanwhile, with the development of fast-growing AI technologies, many promising methodologies are proposed in the healthcare field recently. In this paper, we review and discuss the application of machine learning (ML) methods in health big data in two major aspects: (1) Special features of health big data including multimodal, incompletion, time validation, redundancy, and privacy. (2) ML methodologies in the healthcare field including classification, regression, clustering, and association. Furthermore, we review the recent progress and breakthroughs of automatic diagnosis in health big data and summarize the challenges, gaps, and opportunities to improve and advance automatic diagnosis in the health big data field.

## Introduction

The global “digital divide” *status quo* is quickly changing with the progress in artificial intelligence (AI) technologies and their application area expansion. Nowadays, AI has been widely researched and achieves great success in recent years, and the heart of AI technologies is machine learning (ML) algorithms (Xiong et al., 2022). With the development of the digital economy, Internet, Internet of things (IoT), mobile Internet, and cloud technologies, the application of AI based on health big data presents an explosive increase in recent years (Gokmen and Vlasov, 2016; Dolley, 2018; Ngiam and Khor, 2019; Ye et al., 2021; Weerasinghe et al., 2022). Besides, large amounts of personal health records (PHRs), electronic medical records (EMRs), and electronic health records (EHRs) in hospitals, many governments, and health organizations built the public health monitoring system to collect health data (Heart et al., 2017), such as NEDSS (National Electronic Disease Surveillance System) (National Electronic Disease Surveillance System Working Group, 2001), ProMED-mail (Yu and Madoff, 2004), GPHIN (Global Public Health Intelligence Network) (Dion et al., 2015), HealthMap (Freifeld et al., 2008), MediSys (Linge et al., 2010) and BioCaster (Collier et al., 2008). Among the public health regulatory systems, the representative system is NEDSS. It first defined the standard data protocol to ensure the medical or healthcare data with the identical data format collected across the country. Then, it enables large organizations to upload data automatically through electronic data interchange. The system mainly focused on the collection, exchange, and reporting of diseases and is lagging behind in knowledge mining and early disease warning. Meanwhile, the Internet giants like Google, Facebook, and Twitter collected large amounts of Internet social network data through their products and achieved influenza and other infectious diseases for early warning and tracking (Ginsberg et al., 2009; Signorini et al., 2011; Oflac et al., 2015). Google developed flu outbreak forecast software Google Flu, and the corresponding research result was published in Nature which invoked a large influence on the academic community (Oflac et al., 2015). However, recent research showed that the above-mentioned model in the prediction of flu outbreak existed big defects due to the instability of social network data (Lazer et al., 2014). Intel and IBM companies have also tried to use AI technologies for diabetes control (Nachman et al., 2010; Neuvirth et al., 2011) and the research results were published at the top conference of KDD (Knowledge Discovery in Database) (Neuvirth et al., 2011).

**FIGURE 1:**
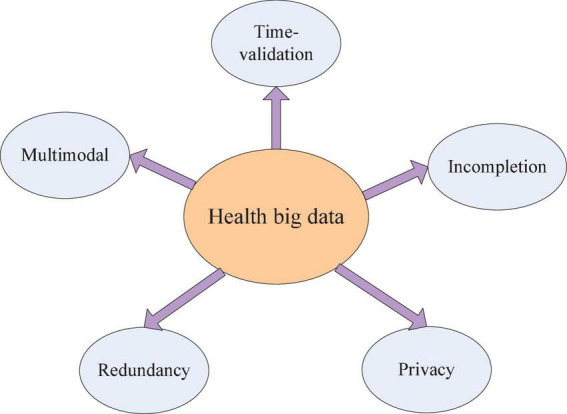
Five special features of health big data.

It is widely accepted that health big data have the potential to help physicians to improve diagnosis and aid drug usage. However, there exist many challenges in processing health big data even though researchers have achieved a lot of good results and applications. Except for five major features (5Vs) Volume, Velocity, Variety, Veracity, and Value, health big data have five additional special features (shown in Figure 1) as follows:

(1) Multimodal: healthcare data consist of text data, image data, and numerical data.

(2) Incompletion: There is a gap between medical data collection and treatment, which cause disease information reflection not enough. At the same time, recording data manually would have deviation, incompletion, and expression uncertainty due to subjective cognition.

(3) Time validation: There is progress between the patient’s treatment and the disease outbreak. For example, electrocardiogram (ECG) and electroencephalogram (EEG) are time signals which have strong time-validated properties.

4) Redundancy: There are many same records stored in the healthcare data system. Take EHRs for example, physicians who serve in community hospitals often input multiple records due to unfamiliar computer operations, especially in China.

5) Privacy: It is inevitably related to the patient’s private information when researchers process healthcare data. Disclosure of patients’ privacy information will hurt patients’ lives.

The rest of this paper is organized as follows. Section II introduces big data technologies like Hadoop, Spark, and Storm. The artificial intelligence technologies in health big data are described in section III. Section IV summarizes this paper.

## Big data technologies

In the big data era, the traditional data management framework which is based on a relational database management system (RDBMS) is challenged by the increasing data deluge. The old framework is unable to deal with the growing amount of unstructured data. Therefore, new technologies were developed to serve the need for big data management, in the aspects of file systems and programming models. These technologies aim at providing scalability as well as fault tolerance, to handle the huge volume and heterogeneity of big data (Wang, 2017). Big data have a wide range of applications, including Smart Grid cases, E-health, Internet of Things, Public utilities, Transportation and logistics, and other areas. The following passages introduce newly developed big data technologies in two aspects in detail.

### Distributed file system

Although Moore’s law promised that the storage capacity of computer chips doubles roughly every 18 months or so, current magnetic storage technology relies on a million atoms per bit, and the quantity of data grows much faster (Bradley, 2017). It is in great demand to develop an efficient and persistent distributed file system. In 2000, Brewer proposed the CAP theorem which states that it is impossible to meet the requirements of consistency, availability, and partition tolerance in an asynchronous distributed read/write system (Wang and Manzie, 2022). As frequent requests are common in big data scenarios, distributed file systems are commonly designed as AP systems, in which only eventual consistency rather than strong consistency is ensured.

**FIGURE 2:**
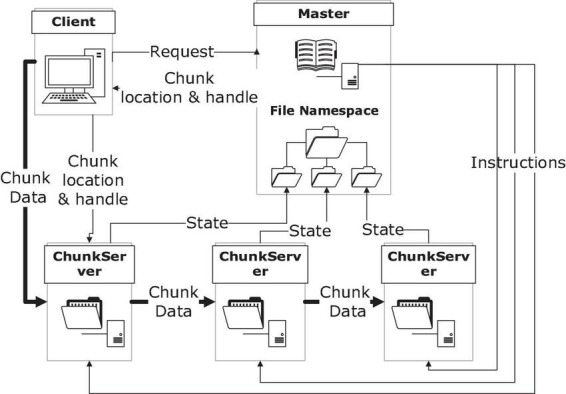
Google file system architecture.

As a pioneer in the attempts of providing users with high-performance services with a distributed file system, Google File System (GFS) achieved great success and its concepts were inherited by a lot of its successors. It features work division between control and storage servers, and replication of the same data, to provide performance and reliability in this way (Ghemawat et al., 2003). Its basic architecture and the data flow in it during a writing procedure was shown in Figure 2.

The highlight of this architecture includes that the control flow is separated from the data flow which leads to higher performance and that the replicas of data offer both reliability and efficiency under good management. Meanwhile, this system also has limitations in supporting small files, for its specific design purpose to support Google’s own service.

Some successors of GFS are generally different implementations of the same idea, for example, HDFS (Kumari and Bucker, 2022) and KosmosFS. Others made some improvements to meet their own demands. Facebook developed Haystack which reduces disk operations for metadata lookups and increases overall throughput to support their Photos application (Beaver et al., 2010). Taobao developed TFS (Fu et al., 2014) which provides significantly higher performance in dealing with small files to support their online shopping service.

In conclusion, after many years of development, distributed file systems are relatively mature, and it is a prevailing trend to develop a customized DFS for a certain field.

### MapReduce framework

As scalability and performance are two of the key requirements of a big data system, parallel computing must be implemented to offer these features. However, traditional parallel programming models fail in migrating to big data systems which consist of a massive number of servers over a wide area. In recent years, a lot of programming models were proposed to provide solutions to this specific need.

**FIGURE 3:**
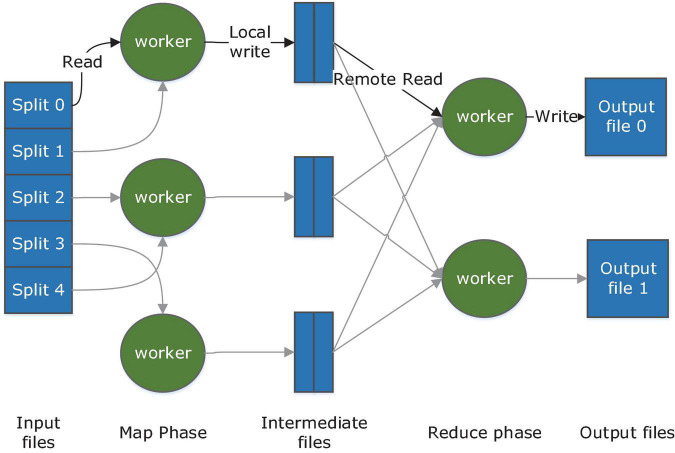
MapReduce work procedure.

As the forerunner in distributing heavy computations across thousands of machines, MapReduce abstracted two basic operations from a broad variety of real-world tasks (Kalia and Gupta, 2021). The map function takes an input pair and produces a set of intermediate key/value pairs which will then be grouped and passed to the Reduce function. Reduce function is responsible for merging a set of values for one key to form a possibly smaller set of values. Once programmers give the proper definition to the two operations, the underlying runtime system will automatically parallelize and distribute the computation and handle other details including machine failures and inter-machine communication. The major part of its work procedure is illustrated in Figure 3.

Many programming models have been proposed afterward. Some provided considerable improvement to the MapReduce model. Microsoft developed Dryad (Isard et al., 2007) in which a job is abstracted as a directed acyclic graph. Each vertex is a program, and data channels are represented by edges. Higher generality is reached as data channels can be customized to support functions more than Map and Reduce. Spark (Solovyev et al., 2010) introduced an abstraction called resilient distributed datasets (RDDs) and parallel operations on them. An RDD represents a read-only collection of objects across a set of machines. By combining parallel operations based on data, Spark avoids redundant I/O operations and multiplied the performance. Other models focus on specific categories of distributed computing. Pregel (Malewicz et al., 2010) aims at large-scale graph processing, in which poor locality of memory access and very little work per vertex often lead to poor efficiency. Storm, as a stream processing model, offers outstanding performance in event processing and incremental computation.

## Artificial intellectual technologies

In the big data era, various public hospitals and private healthcare providers are producing large amounts of data that are difficult to process. Therefore, powerful automatic artificial intellectual algorithms are needed for the analysis and processing of useful information from healthcare data. This information is very precious for healthcare specialists and physicians to apprehend the cause of diseases and for providing better and cost-effective treatment to patients. To improve prediction accuracy, there are various artificial intelligence technologies such as classification, regression, clustering, and association used in healthcare data analysis to increase the healthcare provider’s capability for making the decision in regard to patients health. There are large amounts of research resources available regarding artificial intellectual application in health big data which are presented in subsequent sections with their advantages and disadvantages.

### Classification

**FIGURE 4:**
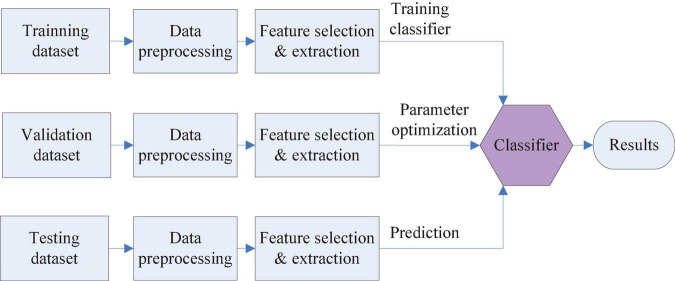
Classification flowchart.

One of the data analysis tasks is classification, which divides data into target labels. Each data point is predicted into the target label by a pattern classifier. For instance, hypertension patients can be classified into three stages of stage 1 hypertension, stage 2 hypertension, and stage 3 hypertension (Wermelt and Schunkert, 2017) on the basis of a supervised classification model. Dataset is often partitioned into a training set, validation dataset, and testing dataset. The training dataset is utilized for training the classifier. The validation dataset is used to tune the classifier parameters to achieve optimal performance. Testing dataset verifies the classification accuracy. Figure 4 shows the entire flowchart of classification.

In the ML domain, SVM as a supervised classifier is widely used for classification (Tsang et al., 2005). It is widely applied in healthcare data recently. Fei proposed the PSO-SVM model which has a strong global search capability (Fei, 2010), and the PSO-SVM model is applied to the diagnosis of arrhythmia cordis, in which PSO is used to determine the free parameters of the support vector machine (Cuong-Le et al., 2022). The testing results showed that the average classification accuracy is 95.65%. Huang et al. (2008) developed a hybrid SVM-based strategy with feature selection to render a diagnosis between breast cancer and fibroadenoma and to find the important risk factor for breast cancer (Azar and El-Said, 2014). The experimental results showed that the features {HSV-1, HHV-8} or {HSV-1, HHV-8, CMV} could achieve identical high accuracy, at 86% of the average overall hit rate. Zheng et al. (2014) used a hybrid of K-means and support vector machine (K-SVM) algorithms to extract useful information and diagnose the tumor. According to 10-fold cross-validation, the developed methodology which was tested on the Wisconsin Diagnostic Breast Cancer (WDBC) dataset from the University of California—Irvine ML repository, increased the accuracy to 97.38%. Avci utilized the genetic-support vector machine (GSVM) approach to classify the Doppler signals of heart valve diseases (Gonzalez-Abril et al., 2014). With the combination of feature extraction and classification from measured Doppler signal waveforms, the performance of the GSVM system showed that this GSVM system is effective to detect Doppler heart sounds. The average rate of correct classification rate was about 95%.

A decision tree (DT) is a common ML method for constructing prediction models from data. The models are obtained by recursively partitioning the data space and fitting a simple prediction model within each partition (Kotsiantis, 2013; Loh, 2014). Due to its results with features of human-readable and interpretable, DT is widely used by many researchers in the healthcare field. Khan et al. (2008) proposed to investigate a hybrid scheme based on fuzzy decision trees, as an efficient alternative to predict breast cancer survivability for personalized healthcare. The experimental results showed that, for cancer prognosis, hybrid fuzz decision tree classification can achieve an average accuracy of 85%. Levashenko et al. (2016) proposed fuzzy decision trees in the medical decision-making support system. The classification accuracy of breast cancer was over 96%. Hassan et al. (2011) developed a decision tree with a CART classification algorithm to forecast response to therapy with 200 chronic hepatitis C patients. The overall classification error was 20%, and 80% was the best accuracy. Moon et al. (2012) developed decision tree models for characterizing smoking patterns in older adults. Their results suggest that social workers need to provide more customized and individualized interventions to older adults. Chang and Chen (2009) applied a decision tree and neural network to increase the quality of dermatologic diagnosis. Using sensitivity analysis combined with the decision tree model, on the contrary, has the least accuracy, which is 80.33%.

A neural network (NN) is based on a biological nervous system having multiple interrelated processing elements known as neurons, functioning in unity to solve a classification problem. Rules extracted from the trained model help to improve the interoperability of the learned network (Schmidhuber, 2015). Er et al. (2010) developed an artificial neural network (ANN) to diagnose chest diseases. Sokolov (2018) presents recent research on approaches in autonomous systems for combining multiple modalities for emotion estimation based on neural networks. Sharma and Parmar (2020) utilized a neural network approach to analyze a heart disease dataset the experimental results proved better accuracy (90.76%) than other optimizations. It is applied to heart disease datasets and finds out a good prediction (Sharma and). In the past several years, intricate neural networks have inspired the further development of intelligent systems. Many disciplines, including the complex field of medicine, neuroimaging modalities, and diagnosis of the disease, have taken advantage of the useful applications of artificial neural networks (Yang et al., 2018; Deperlioglu et al., 2020).

Bayesian decision theory is a basic method under the statistic framework, and it is extended easily to do classification tasks (Chickering et al., 2004). Liu and Lu (2009) proposed to use Bayesian belief network (BBN) as decision support for the higher-level risk estimate which can represent the probabilistic relationships between all kinds of health effects and air pollutants. Dawson et al. (2015) used the Bayesian network to produce the baseline distribution by taking the joint distribution of the data and conditioning it on attributes that are responsible for anomaly pattern detection for disease outbreaks. Curiac et al. (2009) analyzed the psychiatric patient data using BBN in making a significant decision regarding patient health suffering from psychiatric disease and performed an experiment on real data obtained from Lugoj Municipal Hospital.

Long et al. (2015) proposed a heart disease diagnosis system using rough set-based attribute reduction and interval type-2 fuzzy logic system (IT2FLS). The experimental results showed that it could efficiently find minimal attribute reduction from the high-dimensional dataset that enhances the performance of the classification system. The use of an interval type-2 fuzzy logic system for the classification of heart disease datasets to handle the uncertainties and noisiness of these datasets was successful. Nahar et al. (2013) presented the potential of an expert judgment-based (i.e., medical knowledge-driven) feature selection process (termed as MFS). The medical knowledge-based feature selection method has shown promise for use in heart disease diagnostics. The main classification methods used in healthcare big data are shown in Table 1.

**TABLE 1:** Main application of classification methods in healthcare big data.

Method	Scenes	Features
SVM	Diagnosis of arrhythmia cordis; diagnosis between breast cancer and fibroadenoma; diagnosis of the tumor; detect Doppler heart sounds and so on	Non-linear mapping; low generalization error rate, fast classification; suitable for small samples, excellent generalization ability, etc.,
DT	Predict breast cancer survivability; medical decision-making support system; characterizing smoking patterns and so on	Simple to understand, easy to explain, visualization, and wide applicability; prone to overfitting, in addition, small changes in the data can affect the results and are unstable
NN	Including the complex field of medicine, neuroimaging modalities, and diagnosis of the disease; image analysis and interpretation	With self-learning function; no *a priori* assumptions about the problem model are required. suitable for some problems with very complex environmental information, unclear knowledge background, and unclear inference rules.
BN	Anomaly pattern detection for disease outbreaks; regarding patient health suffering from psychiatric disease	Distribution of input data in each layer of the network is relatively stable, which accelerates the model learning speed; makes the model less sensitive to the parameters in the network, simplifies the tuning process, and makes the network learning more stable

The application of classification analysis methods in medicine is getting more and more advanced, not only is it used extensively in disease diagnosis, but also there will be more breakthroughs in disease treatment options in future, and all of these expectations become more apparent in the near future.

### Regression

Regression analysis is a statistical method to determine the quantitative relationship between two or more variables. Based on observational data, regression analysis could establish appropriate dependencies between variables and analyze the inherent rules of data (Merrick et al., 2022). It is widely used for forecasting in the healthcare field. Gutiérrez et al. (2010) proposed a hybrid multi-logistic methodology, named logistic regression using initial and radial basis function (RBF) covariates. Agarwal (2011). developed weighted support vector regression (SVR) approach for remote healthcare monitoring. Vinsnes et al. (2001) developed a regression analysis approach for healthcare personnel’s attitudes to predict nursing assistants’ attitudes. Ko and Osei-Bryson (2004) explored the productivity impact of information technology (IT) in the healthcare industry using a regression spline (RS)-based approach. Luo et al. (2012) presented scalable orthogonal regression (SOR) for non-redundant feature selection and its healthcare applications.

Regression analysis can accurately measure the degree of correlation between factors and the degree of the regression fit to improve the effectiveness of prediction, which is of great significance in medical diagnosis. More recently, regression analysis is one of the most frequently used analytical techniques in disease diagnosis and etiology analysis (Hannan et al., 2010; Liu et al., 2018; Jfri et al., 2021).

### Clustering

Clustering is an unsupervised learning method that is different from classification. Clustering is a process of classifying data into different classes or clusters, so objects in the same cluster have a large similarity, and objects between different clusters have a large degree of dissimilarity (Caron et al., 2018). Clustering is also used in the healthcare field. Sinaga and Yang (2020) proposed a novel unsupervised k-means (U-k-means) clustering algorithm which automatically finds an optimal number of clusters without giving any initialization and parameter selection. Stein et al. (2007) used data clustering techniques to develop health state descriptions based on data from 66 women who completed the EORTC QLQ-C30 over a 6-month period while receiving chemotherapy for ovarian cancer. Belciug et al. (2010) detected breast cancer recurrence with the help of a clustering-based approach. Zhao et al. (2020) proposed to propose a new deep learning and clustering UDFCMN (Unsupervised Deep Fuzzy C-Means clustering Network) model, to cluster lung cancer patients from lung CT images; these results also indicate that this method has practical applications in lung cancer pathogenesis studies and provide useful guidelines for personalized cancer therapy. Balasubramanian and Umarani (2012) analyzed the impact of fluoride on human health (dental) with the help of a clustering-based method and found meaningful hidden patterns which gave meaningful decision-making to this socio-economic real-world health hazard. In addition, some researchers have also used clustering methods to early detect Alzheimer’s disease (Escudero et al., 2011; Holilah et al., 2021). The main clustering methods used in healthcare big data are shown in Table 2.

**TABLE 2:** Main application of clustering methods in healthcare big data.

Method	Scenes	Features
k-means	Health state descriptions; Alzheimer’s disease; health hazards and so on	Fast convergence; better clustering effect; stronger interpretability of the model and so on
Fuzzy C-means	Lung cancer patients from lung CT images	Clustering objectively and accurately

Cluster analysis is essentially finding a statistic that objectively reflects the affinity of an element and then classifying the elements into categories based on this statistic. Cluster analysis decomposes the symptoms of chronic diseases and is used to assess the quality of life in chronic diseases, such as lung cancer; cluster analysis is very effective in assessing these diseases.

### Association

Association is one of the most vital approaches to data mining that is used to find out the frequent patterns, and interesting relationships among a set of data items in the data repository. Frequent patterns are patterns that appear frequently in a dataset. The initial motivations of the association rules were raised for the issue of the market basket analysis. The association process analyzes the customer’s shopping habits by discovering the association between the different items placed in the “shopping basket” by the customer. The discovery of this association can help retailers understand which goods are frequently purchased by customers at the same time, so as to help them develop better marketing strategies (Tomar and Agarwal, 2013). Association also has a great impact in the healthcare field to detect the relationships among diseases, health status, and symptoms. Nahar et al. (2013) presented a rule extraction experiment on heart disease data using different rule-mining algorithms (*Apriori*, Predictive *Apriori*, and Tertius). Further rule-mining-based analysis was undertaken by categorizing data based on gender, and significant risk factors for heart disease were found for both men and women. Ji et al. (2010) developed a new interestingness measure, exclusive causal-leverage, based on an experience-based fuzzy recognition-primed decision (RPD) model. On the basis of this new measure, a new association rule algorithm is proposed to discover infrequent causal relationships in electronic health databases (Horton et al., 2019). In addition, Soni and Vyas (2010) used the associative method to construct a classifier for predictive analysis in healthcare data mining.

Using the association analysis method to discover the relationship between the attributes in the medical dataset, especially some general factors such as age and smoking habits, and the measured body organ function indices related to the possibility of disease, the doctor can accurately determine the possibility of disease through the patient’s characteristics, which is very meaningful for medical diagnosis, and the future application of the association analysis method to predict diseases and develop treatment plans based on vital signs.

In summary, AI as a role in healthcare big data, its effects on the development of the medical industry, applications of AI in medicine, challenges, and promises of both AI and big data with respect to healthcare, and prevailing techniques (methods such as deep neural network, convolutional neural network, and recurrent neural network) and tools for performance optimization of healthcare big data can be used by the medical industry.

## Conclusion

This paper investigated the application of AI technologies in health big data based on a data elements perspective. Traditional data management framework, which is based on a relational database management system (RDBMS), is hard to deal with a growing amount of healthcare data. Big data processing frameworks like Hadoop (Spark et al.) are employed in the data preprocess stage to accommodate big data and accelerate computing efficiency. We found that there is no single ML method that gives consistently good results for all kinds of health big data. The performance of ML methods depends on the type of dataset that researchers have taken for doing the experiment. To get the higher performance of ML method, most of the research combined many artificial methods to complement the deficiency of each one called hybrid method or integrated method or assemble method.

In addition, it is well known in the AI field that feature selection and extraction are very important factors that affect the performance of artificial methods. Features are extracted and selected on the basis of healthcare/medical domain knowledge and optimal techniques in normal conditions. For healthcare providers and medical providers, AI technologies are widely utilized to make effective decisions in regard to how to enhance patients’ health, how to provide healthcare service at low cost, and how to remind physicians to avoid misusing drugs and misdiagnosing.

## Data availability statement

The original contributions presented in this study are included in the article/supplementary material, further inquiries can be directed to the corresponding author/s.

## Author contributions

HX analyzed the methods and penned the manuscript. HC, LX, and HL gave important suggestions. QT, LF, and HFC participated in discussions and provided some literature resources. HC revised the manuscript. All authors contributed to the article and approved the submitted version.
